# Interfering Plasmons in Coupled Nanoresonators to Boost Light Localization and SERS

**DOI:** 10.1021/acs.nanolett.0c04987

**Published:** 2021-03-11

**Authors:** Angelos Xomalis, Xuezhi Zheng, Angela Demetriadou, Alejandro Martínez, Rohit Chikkaraddy, Jeremy J. Baumberg

**Affiliations:** † NanoPhotonics Centre, Cavendish Laboratory, Department of Physics, University of Cambridge, JJ Thompson Avenue, Cambridge CB3 0HE, United Kingdom; ‡ Department of Electrical Engineering (ESAT-TELEMIC), KU Leuven, Kasteelpark Arenberg 10, BUS 2444, 3001 Leuven, Belgium; § School of Physics and Astronomy, University of Birmingham, Birmingham B15 2TT, United Kingdom; ∥ Nanophotonics Technology Center, Universitat Politècnica de València, Valencia 46022, Spain

**Keywords:** Nanocavity, field enhancement, near-field, SERS, nano-optics, plasmon interference, remote excitation

## Abstract

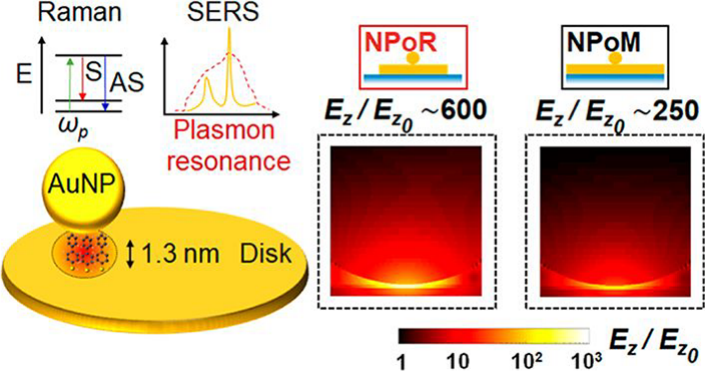

Plasmonic self-assembled nanocavities are ideal platforms for extreme light localization as they deliver mode volumes of <50 nm^3^. Here we show that high-order plasmonic modes within additional micrometer-scale resonators surrounding each nanocavity can boost light localization to intensity enhancements >10^5^. Plasmon interference in these hybrid microresonator nanocavities produces surface-enhanced Raman scattering (SERS) signals many-fold larger than in the bare plasmonic constructs. These now allow remote access to molecules inside the ultrathin gaps, avoiding direct irradiation and thus preventing molecular damage. Combining subnanometer gaps with micrometer-scale resonators places a high computational demand on simulations, so a generalized boundary element method (BEM) solver is developed which requires 100-fold less computational resources to characterize these systems. Our results on extreme near-field enhancement open new potential for single-molecule photonic circuits, mid-infrared detectors, and remote spectroscopy.

## Introduction

Localization of light in hotspots far smaller than the incident wavelength is one of the key advantages of metallic cavities over their dielectric counterparts. Using such localization to guide and confine light at the nanoscale is beneficial for technologies including photovoltaics,^1^ integrated waveguides,^2^ photodetectors,^3^ lasers and amplifiers,^4,5^ and biological imaging,^6^ as well as underpinning nanophotonics research.

Squeezing light into small mode volumes enhances light–matter interactions dramatically, allowing even single-molecule spectroscopies.^7−9^ Metal–insulator–metal (MIM) nanocavities, formed by nanogaps between self-assembled metal building blocks, comprise relative low *Q*-factors (∼10–30) but extremely small mode volumes *V*_m_ < 50 nm^3^, resulting in Purcell factors (*Q*/*V*_m_) exceeding ∼10^6^ (for insulating gaps <2 nm). Such plasmonic nanocavities can thus facilitate strong light–matter interactions under ambient conditions, enhanced emission rates, and high radiative quantum efficiency^10−14^ (for gaps >0.5 nm as used here, quantum spill-out and tunnelling effects have only minor effects). One type of MIM cavity that has attracted much recent interest is the nanoparticle-on-mirror (NPoM) geometry where a plasmonic nanoparticle is spaced by a single self-assembled molecular monolayer (SAM) from a metallic mirror.^15^ This fixes the plasmonic cavity gap width at the subnanometer scale and results in field enhancements exceeding *E*/*E*_0_ > 200, which leads to 10^8^-fold intensity enhancement of two photon absorption^16^ and optomechanical nonlinearities.^17^

Recent work^18−20^ suggests the desirability of combining such plasmonic nanocavities with mid-infrared resonators that simultaneously allow access to molecular vibrational absorption as well as the near-infrared plasmon modes for SERS. Both anti-Stokes Raman and surface-enhanced infrared absorption (SEIRA) then become possible, however few structures yet support both techniques. Here we use a nanoparticle-on-resonator (NPoR) construct where metallic disks supporting infrared resonances^21−24^ are coupled to nanoparticles (of much smaller radius) to form NPoM nanocavities. We show that such structures support resonances in the visible regime. However, a subtle interplay of different optical couplings has to be understood to interpret the scattering resonances and SERS spectra on disk diameters *D* = 1–6 μm. We find that light is coupled into high-order modes on the disk, both via the disk edges and via the nanoparticle, thus allowing additional levels of field enhancement.

In this work we use simulation results to analyze and interpret experimental results for the enhanced SERS observed. Because of the computational demands of simulations which combine subnanometer gaps with >5 μm disks (discretization >10^10^ elements), we use a more generalized boundary element method (BEM) solver. This method uses a potential-based formalism that can model local and hydrodynamic nonlocal responses and, thus, is ideal for plasmonic nanoconstructs like the one studied here, as well as waveguides.^25,26^ To confirm its reliability, we compare finite-difference time-domain simulations (FDTD) with our new BEM solver. In contrast to the FDTD algorithm where the entire simulation volume is discretized, the BEM solver only discretizes the boundary of the nanoscatterers. Consequently, the BEM method demands 100-fold less computational resources and gives tractable computational speeds compared to its FDTD counterpart (as well as finite element methods (FEM), and other electromagnetic computational techniques). This advance is crucial for nanophotonic devices that span wavelengths from 0.5 to 15 μm and sizes from 0.1 nm to 10 μm.

## Results and Discussion

**Figure 1:**
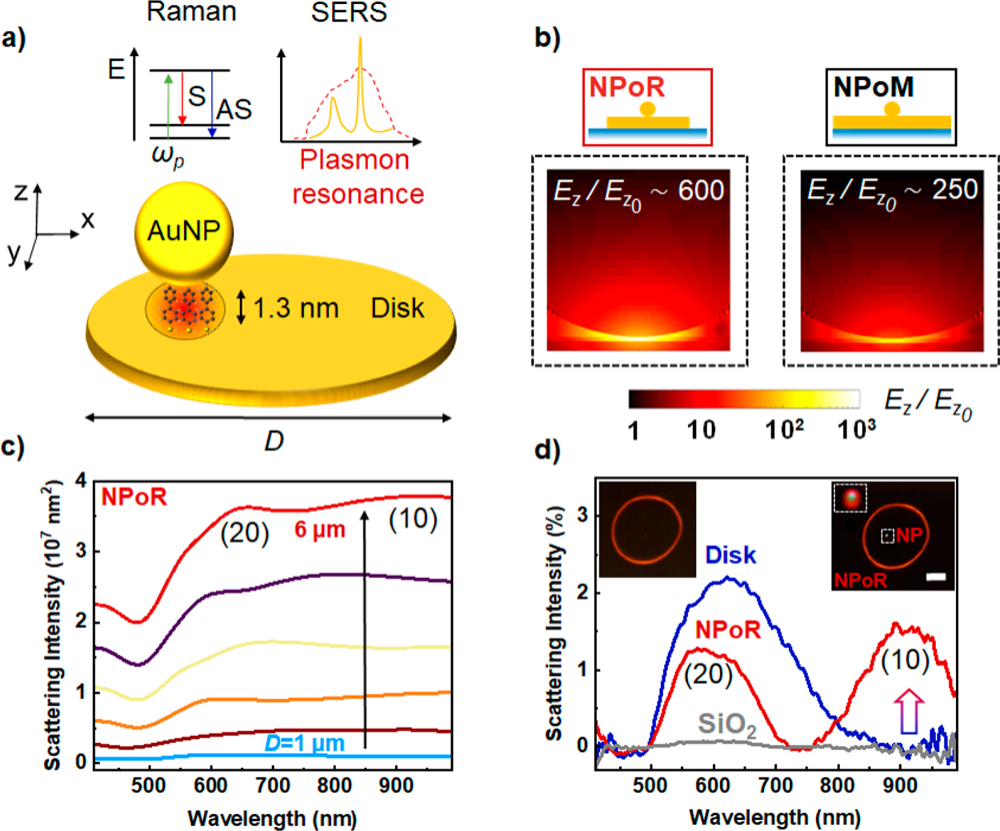
Near- and far-field plasmon resonances of the nanoparticle-on-resonator (NPoR) construct. (a) NPoR configuration with a 100 nm Au disk thickness and self-assembled monolayer (SAM) of biphenyl-4,4′-dithiol (BPT) creating a dielectric spacer set by the molecule length (1.3 nm). Insets show the Raman process and plasmonic SERS amplification. (b) Simulated near-field maps of NPoR and NPoM for λ = 750 nm light incident at 52°. (c) BEM simulations of total scattering intensity from NPoR for increasing disk diameters, *D* = 1–6 μm. (d) Experimental dark-field spectra of bare disk (dark blue), NPoR (red), and SiO_2_ substrate (gray). Inset shows DF images of empty disk and NPoR. Arrow shows (10), the main nanocavity resonance. The disk diameter is 6 μm.

We exploit a plasmonic system that is capable of large-scale deployment with robust reliable plasmonic enhancements.^21^ We combine bottom-up assembly of nanocavities with top-down photolithography that forms the disk resonator. This is achieved by placing Au nanoparticles (80 nm diameter) on top of a 100 nm-thick disk microresonator (μ-resonator) of variable diameter (Figure 1a). The nanocavity gap can be controlled at the subnanometer scale using a dielectric molecular spacer self-assembled on top of the Au disk (see Methods). At optical frequencies, induced dipoles in the Au nanoparticle couple to their image charges in the underlying disk, delivering tight field confinement similarly to spherical dimers.

We concentrate here on metallic disks 6 μm in diameter with array modes around λ = 10 μm (see Section S1) and possessing higher-order resonances in the vis/NIR (Section S4). Although the disk edges are far away from the NPoM, we find that the disk μ-resonator amplifies the initial near-field confinement in the NPoM to boost the SERS intensity, *I*_SERS_ ∝ [*E*_tot_(λ_in_)]^2^[*E*_tot_(λ_out_)]^2^. Specifically, we show how the interplay of the two resonators and their relative position delivers a 3-fold enhancement of the near-field in the gap, amplifying the SERS signal. In-coupling at the disk edges launches high-order modes into the NPoM gap and can remotely excite the embedded molecules in the nanocavity.

Metal–insulator–metal nanoparticle-based cavities give strong scattering resonances which depend on the gap and nanoparticle facet size.^15,27,28^ The near-field BEM simulations show a 3-fold enhancement for the NPoR compared to NPoM constructs under plane-wave excitation at an optimal incident angle of 52° (Figure 1b). BEM simulations also show the increasing scattering intensity as the diameter of the disk increases from 1 to 6 μm (Figure 1c), as expected from their relative areas and becoming >100-fold larger than the NPoM (see Figure S3.2). To decipher the overall scattering response of the NPoR we perform dark-field measurements, exciting and collecting light tightly focused on different locations (Figure 1d, insets). These measurements clearly distinguish between the scattering of the bare disk and the overall NPoR structure (Figure 1d). The (10) nanocavity resonance of the NPoR (red arrow) is absent in the bare disk (dark blue). The (*lm*) indices for identifying nanoparticle resonances are in accord with previous work^27^ and label the radial and azimuthal near-field symmetries.

**Figure 2:**
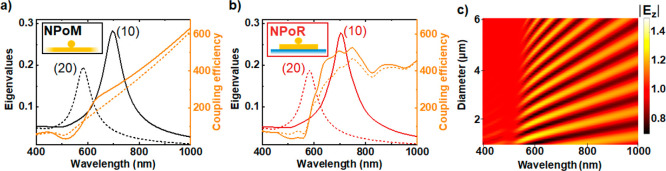
Enhanced light localization in nanocavities driven by an external resonator. (a, b) Eigen-analysis of (a) NPoM and (b) NPoR plasmonic constructs. Eigenmodes of NPoM (black) and NPoR (red) and coupling efficiency (orange) for (20) (dashed) and (10) (solid) resonances. Insets show each plasmonic system. (c) Total electric field *E*_*z*_ from outward and reflected plasmons at the center of the disk for increasing disk diameters, *D* = 1–6 μm.

To better examine this field enhancement, we apply separate eigenmode analysis for the NPoR and NPoM plasmonic constructs.^29^ Both systems support (20) and (10) states (full eigenmode analysis in Section S3). This analysis is helpful since eigenmodes and eigenvalues are independent of excitation conditions,^30^ while the eigenvalue magnitude calibrates the enhancement of each corresponding eigenmode (evaluating *Q*-factors requires separately solving the natural modes or quasi-modes of the system^31^). Similar eigenvalues for both NPoR and NPoM indicate that they support similar resonances (Figure 2a,b), since for large disk diameters (*D* = 6 μm) compared to vis–NIR wavelengths, the disk behaves similarly to the infinite mirror in the NPoM. By contrast, the (10) coupling efficiency is 50% higher for NPoRs than for NPoMs (orange, Figure 2a,b) at the resonance. As we show below, this higher coupling efficiency is driven by the high-order modes of the disk resonator. For wavelengths below 550 nm, strong Au absorption attenuates the plasmon disk resonances resulting in similar (20) coupling efficiencies for NPoMs and NPoRs (dashed orange, Figure 2a,b). To investigate the importance of the high-order disk modes in near-field enhancement, we plot the electric field (*E*_*z*_) at the center of a bare disk resonator which is the sum of outward and reflected disk plasmons (Figure 2c).^32^ For increasing disk diameters *D* = 1–6 μm, more interfering modes appear in the visible regime (see Section S4), modulating the field under the nanogap. Combining nanocavity resonances with high-order modes of the microdisk thus boosts light localization in the gap region (Figure 1b).

**Figure 3:**
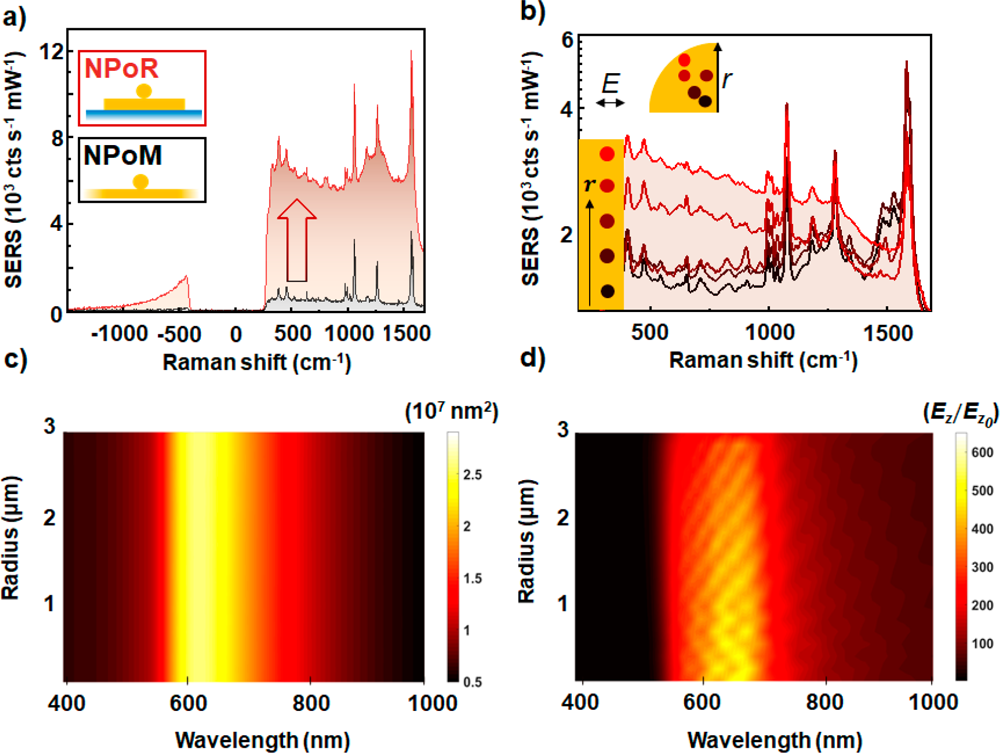
SERS amplification in nanocavities embedded in a μ-resonator. (a) SERS measurements in NPoR (red) and NPoM (black) geometries, averaged over 20 Au nanoparticles in each case. (b) Spatial dependence of SERS, for increasing radial positions (*r*) of nanoparticles from the disk center (as colored in the inset). (c, d) BEM calculations of the (c) total scattering intensity and (d) near-field in the nanogap of a nanoparticle placed at increasing radial positions on the disk. Light excitation at 52° onto 80 nm Au NP on a 6 μm diameter disk.

To understand this spatial dependence, we explore the near-field response using SERS from the biphenyl-4,4′-dithiol (BPT) molecules through systematic measurements on 40 particles at different distances *r* from the disk center. These are directly compared to NPoMs prepared under identical conditions (see Methods). A 633 nm laser of 1 mW is tightly focused with an 0.8 NA objective lens onto each nanoparticle. The average SERS intensity of BPT vibrational peaks on NPoRs is ∼200% larger than in NPoMs (comparing background-subtracted peaks), as predicted from the higher local optical field (Figure 3a). This enhancement varies with the vibration energy (and hence emission wavelength). Even more evident, NPoRs show a much higher SERS background which is known to mostly arise from electronic Raman scattering (ERS) when light (in the form of plasmons) penetrates inside the metal.^33^ The overall ERS enhancement in NPoRs is >1000%, with extra resonances apparent around 1250 cm^–1^ (687 nm) of spectral width 200 cm^–1^ (9 nm).

Examining the field profiles for the high-order disk modes and NPoM modes shows that SERS originates only from the NPoM gap, while ERS arises both in the gap and from SPP modes propagating to the disk edges. The nanoparticle contribution of enhancement in both SERS and ERS signals from the increased local gap fields are the same, and hence, the extra ERS arises from the high-order modes of the disk. The visibility of the mode interference fringes versus disk diameter in simulations suggests round trip losses of ∼30%. In-coupled light scatters from the NP into high-order modes which bounce off the disk edges and return to the NPoM now containing additional scattered ERS light. We can thus quantify both the NPoM coupling as well as the strength of the returning high-order mode light, which depend on the exact geometry of the nanoparticle facet as well as the shape of the disk edge (see Section S4).

To check this dependence, we plot the SERS spectra for nanoparticles positioned in different radial positions from the disk center (Figure 3b). Nanoparticles situated near the disk edge show a higher SERS ERS background (bright red) in contrast to nanoparticles near the disk center (dark red). The shape of this ERS background also varies, while the SERS peaks are found to increase to a maximum ∼50% larger at about halfway out while at the edge they reduce substantially.

To better quantify these fluctuations, we calculate the total scattering and near-field in the gap for a nanoparticle placed in different radial positions on the disk with the BEM solver. Briefly, the surface integral equations are based on the Poggio–Miller–Change–Harrington–Wu formalism and discretized using Rao–Wilton–Gilson basis functions (see Section S3).^34−36^ The total scattering is dominated by a disk resonance at 620 nm and remains unchanged with NP position (Figure 3c). By contrast, light localization inside the NP gap shows an oscillatory behavior with the NP position, resulting from interference of incident and backscattered high-order modes over the μ-resonator surface (Figure 3d). In principle, we expect similar trends in the experimental data; however, we note that a quantitative fit is precluded by our lack of spatial precision due to random nanoparticle positions and the slightly different shapes of each disk (Figure 3b). However, this data confirms the capability to combine mid-infrared disk resonators with visible/NIR nanocavities possessing enhanced optical field coupling.

**Figure 4:**
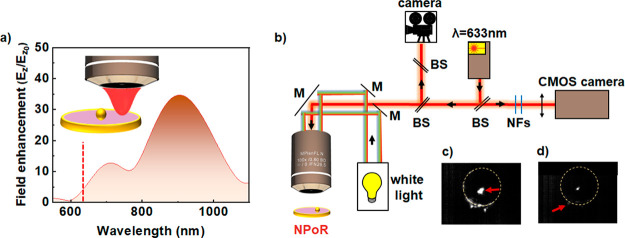
Remote-excitation of molecules in nanocavities using delocalized plasmon modes in a μ-resonator. (a) Simulated electric-field confinement in the gap for 0.8 NA Gaussian excitation onto the disk edge. The red dashed line shows the excitation wavelength (633 nm, 2 mW μm^−2^). (b) Optical setup for remote excitation of molecules in nanocavities. Laser light is blocked by two notch filters (NFs) placed before CMOS camera. (c, d) SERS images from NPoR when laser light excites (c) the nanoparticle directly or (d) the disk edge. Excitation points are marked with red arrows.

The coupling to high-order modes also suggests that remote SERS excitation of NPoMs over several micrometers is possible. This possibility has been suggested for high-resolution SERS imaging,^37−40^ fluorescence microscopy, and catalytic driven reactions.^41^ Accessing molecules inside nanocavities remotely can also prevent molecular damage due to high pump intensities and/or heating. To better quantify the delocalized plasmon modes on the surface of the μ-resonator, we perform additional simulations (FDTD, Lumerical). We consider a disk resonator with a 6 μm diameter and 80 nm Au particle placed exactly at the center on a thin dielectric spacer (1.3 nm) to form a nanocavity. We assume 0.8 NA Gaussian excitation onto the disk edge and monitor the field confinement in the gap (Figure 4a). We see a field localization of *E*/*E*_0_ > 10 for longer visible and near-infrared wavelengths.

To confirm these theoretical findings, we performed a remote SERS experiment. A nanoparticle positioned close to the disk center is first illuminated directly with the 633 nm pump laser. Elastically and inelastically scattered light is collected through the same ×100 0.8 NA objective, filtered through two notch filters, and imaged on a CMOS camera (Figure 4b). The images clearly show that SERS originates mainly from the nanoparticle and disk periphery while all other areas remain dark (Figure 4c, d). This demonstrates that incoming photons can be efficiently coupled into the nanocavity and SERS harvested from all emerging modes. The high-order modes propagate radially out from a nanoparticle launch point (red arrow) and SERS out-scatters from the disk edge (Figure 4c). Such a plasmonic system should obey Lorentz reciprocity, so instead launching plasmons at the disk edge (red arrow, Figure 4d) results in the nanoparticle outcoupling SERS. These SERS intensities differ because of the different in- and out-coupling efficiencies of the nanoparticle and disk edge as well as their different collection efficiencies in the far-field (Figures 1b and 4a). Dark-field images confirm that indeed light is more efficiently scattered from the disk periphery and the nanoparticle, while all other areas remain dark (see inset Figure 1d).

We show light confinement of NPoRs with near-fields *E* up to 3× higher compared to NPoMs, which should boost SERS intensities since *I*_SERS_ ∝ *E*^4^. To quantify this, we consider near-field ratios at the excitation wavelength which is detuned from the spectral position of maximum field. For pump λ = 633 nm, *E*_NPoR_/*E*_NPoM_ ∼ 1.5 (see Section S2) which corresponds to a SERS intensity contrast of 4.7, comparing well to the average experimental ratio of 3.5 (1.8 with background subtraction). One reason this may be lower than expected is that the experimental SERS signal of NPoRs are averaged over 20 nanoparticles randomly positioned on different disks. Field confinement depends strongly on the radial location of the nanoparticle on the μ-resonator (Figure 3d). Current nanoparticle deposition uses simple drop casting onto BPT-coated resonators, resulting in random positioning. This issue may be addressed by lithography, DNA origami-based assembly, or direct optical printing of colloidal nanoparticles onto the disks.^42−45^

We study μ-resonators with mid-infrared resonances (see Section S1) which also support high-order resonances in the vis/NIR. We note that for the current disk design we expect plasmon-enhanced Stokes SERS since the disk resonances are red-shifted from the pump (dashed line Figure 4a).^46^ With 80 nm nanoparticles the (10) resonance is red-shifted from the 633 nm pump^47^ resulting in amplification of Stokes and suppression of anti-Stokes SERS. Future investigations that target the anti-Stokes emission can be optimized by using nanoparticle diameters of 40–60 nm as well suitably tuned pump excitation wavelengths.

Thin disk geometries facilitate a plethora of high-order modes on their surface that amplify near-field enhancements. This allows for random positioning of a nanoparticle on the disk without much reduction in SERS from lower near-field enhancements. Further optimization should explore reduced disk sizes as we find that smaller diameters create more-uniform amplification at frequencies close to the pump (Figure S4).

## Conclusions

We demonstrate that effective optical coupling of a micrometer-scale disk resonator and nanometer-scale molecular gap cavity leads to enhanced light localization (*E*^2^/*E*_0_^2^ > 10^5^). This provides new possibilities in plasmon-based spectroscopies with vibrational peaks of molecules giving >200% higher SERS intensity (>10000% predicted under fully optimized conditions) and a 10-fold stronger electronic scattering compared to standard plasmonic constructs used previously. The superposition of higher-order modes of the micro-resonator with the optical resonances of self-assembled nanocavities is found to control the near-field resulting in modulation of SERS intensities with nanoparticle location. The launching and detection of plasmons from a point which reflects off the disk edge resembles the SNOM experiments that scatter light into surfaces modes.^48,49^ However here we are able to directly measure the near-field enhancements using the molecular SERS signatures. We also show that nanocavities can be accessed remotely via propagating modes over a few micrometers, which can be helpful for preventing molecular damage occurring when molecular monolayers are excited directly.

This dual resonator approach can be extended to higher-Q optical micro-resonators that address specific vibrational modes to Stokes or anti-Stokes sides. Besides MIM structures, there may also be interest in using metal–insulator–dielectric nanocavities, particularly in the mid-IR region. Strong enhancements would thus open access to a wider range of molecules previously ignored due to their low Raman cross sections. Such combinations of molecular nanocavities and plasmonic microresonators may find beneficial uses for optimum light trapping^50^ and low-cost infrared detection.^18^

## Methods

### Sample Preparation: Photolithography and μ-Resonator Fabrication

The SiO_2_ substrates were spin coated with Ti Prime, which acts as an adhesion layer, at 3000 rpm for 20 s and 1000 rpm acceleration. Ti Prime-coated substrates were then placed onto a hot plate and baked for 120 s at 120 °C. Next, 1 μm of a positive photoresist (AZ MIR 701 29CP, MicroChemicals) was spin coated (4000 rpm for 30 s and 1000 rpm acceleration) and later soft-baked at 90 °C for 90 s. For photolithography, we used a fully customized laser printer with a laser source at a wavelength of 375 nm (ProtoLaser LDI, LPKF). A 100 × 100 μm^2^ array was exposed, resulting in disk patterns of 6 μm in diameter. We developed the exposed area by immersing the structures into deionized (DI) water and sonicating them for 25 s gently. Further, we deposited 100 nm of Au with the aid of an E-beam evaporator (at 0.5 nm/s, Kurt J. Lesker). To lift-off the Au-coated resist, we dipped the samples in an acetone solution for 2 h and left the samples to dry, resulting in an array of 100 nm thick Au disk μ-resonators on SiO_2_ substrates.

### Self-Assembly Nanocavities

To create nanometer-scale cavities, we used bottom-up molecular nanoassembly. To do this, the disk μ-resonators were immersed in 1 mM biphenyl-4-thiol (BPT, Sigma-Aldrich, 97%) solution in anhydrous ethanol (Sigma-Aldrich, <0.003% H_2_O) for 12 h. BPT forms a SAM of 1.3 ± 0.1 nm directly on the disks through Au–S bonding. Further, citrate-capped 80 nm Au NPs (BBI Solutions) were deposited by drop casting on the BPT-coated Au-disks. The deposition time was about 20 s (depends on the NP density). The excess NPs were flushed with DI water, and the samples were left to dry.

### Spectroscopy

Elastic (dark-field) and inelastic light scattering (SERS) measurements were performed using a modified optical microscope (Olympus BX51) setup similar to that in ref (47). Briefly, NPoRs were placed on a motorized stage (Prior Scientific H101) which was controlled by in-house Python code. A halogen lamp was used for the dark-field and a spectrally filtered 632.8 nm diode laser (70 mW, Matchbox, Integrated Optics) for SERS measurements with the aid of a long working distance objective lens (×100 0.8 NA). For SERS, the laser light was filtered with a pair of notch filters (633 ± 2 nm, Thorlabs), which was then focused with the aid of a tube lens into the spectrograph (Andor Shamrock i303) and a Newton EMCCD camera. For dark-field spectroscopy, the reflected light from the sample was collected through the same high NA objective and split into an imaging camera (Lumenera Infinity3-1) and a fiber-coupled spectrometer (Ocean Optics QEPRO). For remote SERS experiments, inelastically scattered light from the sample was filtered with a pair of notch filters at 633 nm, expanded, and collimated on a high-resolution CMOS camera (Prime BSI, Teledyne Photometrics).

## Supporting Information Available

Mid-infrared characterization of the disk array, FDTD simulations of different excitation sources, description of the BEM computational method, interfering resonances on the disk surface, and field enhancement in metallic and semimetallic nanocavities (PDF)

## Notes

Source data can be found at DOI link: https://doi.org/10.17863/CAM.65547.

## References

[ref1] Atwater H. A.; Polman A. Plasmonics for improved photovoltaic devices. Nat. Mater. 2010, 9, 205–213. 10.1038/nmat2629. 20168344

[ref2] Maier S. A.; Kik P. G.; Atwater H. A. Observation of coupled plasmon-polariton modes in Au nanoparticle chain waveguides of different lengths: Estimation of waveguide loss. Appl. Phys. Lett. 2002, 81, 1714–1716. 10.1063/1.1503870.

[ref3] Stewart J. W.; Vella J. H.; Li W.; Fan S.; Mikkelsen M. H. Ultrafast pyroelectric photodetection with on-chip spectral filters. Nat. Mater. 2020, 19, 158–162. 10.1038/s41563-019-0538-6. 31768011

[ref4] Berini P.; De Leon I. Surface plasmon–polariton amplifiers and lasers. Nat. Photonics 2012, 6, 16 10.1038/nphoton.2011.285.

[ref5] van Beijnum F.; van Veldhoven P. J.; Geluk E. J.; de Dood M. J. A.; Hooft G. W.; van Exter M. P. Surface plasmon lasing observed in metal hole arrays. Phys. Rev. Lett. 2013, 110, 206802 10.1103/PhysRevLett.110.206802. 25167437

[ref6] Jain P. K.; Lee K. S.; El-Sayed I. H.; El-Sayed M. A. Calculated absorption and scattering properties of gold nanoparticles of different size, shape, and composition: applications in biological imaging and biomedicine. J. Phys. Chem. B 2006, 110, 7238–7248. 10.1021/jp057170o. 16599493

[ref7] Anker J. N.; Hall W. P.; Lyandres O.; Shah N. C.; Zhao J.; Van Duyne R. P. Biosensing with plasmonic nanosensors. Nat. Mater. 2008, 7, 442–453. 10.1038/nmat2162. 18497851

[ref8] Zijlstra P.; Paulo P. M.; Orrit M. Optical detection of single non-absorbing molecules using the surface plasmon resonance of a gold nanorod. Nat. Nanotechnol. 2012, 7, 379–382. 10.1038/nnano.2012.51. 22504707

[ref9] Nie S.; Emory S. R. Probing single molecules and single nanoparticles by surface-enhanced Raman scattering. Science 1997, 275, 1102–1106. 10.1126/science.275.5303.1102. 9027306

[ref10] Chikkaraddy R.; De Nijs B.; Benz F.; Barrow S. J.; Scherman O. A.; Rosta E.; Demetriadou A.; Fox P.; Hess O.; Baumberg J. J. Single-molecule strong coupling at room temperature in plasmonic nanocavities. Nature 2016, 535, 127–130. 10.1038/nature17974. 27296227 PMC4947385

[ref11] Hoang T. B.; Akselrod G. M.; Argyropoulos C.; Huang J.; Smith D. R.; Mikkelsen M. H. Ultrafast spontaneous emission source using plasmonic nanoantennas. Nat. Commun. 2015, 6, 7788 10.1038/ncomms8788. 26212857 PMC4525280

[ref12] Sidiropoulos T. P.; Röder R.; Geburt S.; Hess O.; Maier S. A.; Ronning C.; Oulton R. F. Ultrafast plasmonic nanowire lasers near the surface plasmon frequency. Nat. Phys. 2014, 10, 870–876. 10.1038/nphys3103.

[ref13] Russell K. J.; Liu T.-L.; Cui S.; Hu E. L. Large spontaneous emission enhancement in plasmonic nanocavities. Nat. Photonics 2012, 6, 459–462. 10.1038/nphoton.2012.112.

[ref14] Akselrod G. M.; Argyropoulos C.; Hoang T. B.; Ciracì C.; Fang C.; Huang J.; Smith D. R.; Mikkelsen M. H. Probing the mechanisms of large Purcell enhancement in plasmonic nanoantennas. Nat. Photonics 2014, 8, 835 10.1038/nphoton.2014.228.

[ref15] Baumberg J. J.; Aizpurua J.; Mikkelsen M. H.; Smith D. R. Extreme nanophotonics from ultrathin metallic gaps. Nat. Mater. 2019, 18, 668–678. 10.1038/s41563-019-0290-y. 30936482

[ref16] Ojambati O. S.; Chikkaraddy R.; Deacon W. M.; Huang J.; Wright D.; Baumberg J. J. Efficient Generation of Two-Photon Excited Phosphorescence from Molecules in Plasmonic Nanocavities. Nano Lett. 2020, 20, 4653–4658. 10.1021/acs.nanolett.0c01593. 32422048 PMC7366501

[ref17] Lombardi A.; Schmidt M. K.; Weller L.; Deacon W. M.; Benz F.; de Nijs B.; Aizpurua J.; Baumberg J. J. Pulsed Molecular Optomechanics in Plasmonic Nanocavities: From Nonlinear Vibrational Instabilities to Bond-Breaking. Phys. Rev. X 2018, 8, 011016 10.1103/PhysRevX.8.011016.

[ref18] Roelli P.; Martin-Cano D.; Kippenberg T. J.; Galland C. Molecular Platform for Frequency Upconversion at the Single-Photon Level. Phys. Rev. X 2020, 10, 031057 10.1103/PhysRevX.10.031057.

[ref19] Chen K.; Adato R.; Altug H. Dual-Band Perfect Absorber for Multispectral Plasmon-Enhanced Infrared Spectroscopy. ACS Nano 2012, 6, 7998–8006. 10.1021/nn3026468. 22920565

[ref20] Adato R.; Altug H. In-situ ultra-sensitive infrared absorption spectroscopy of biomolecule interactions in real time with plasmonic nanoantennas. Nat. Commun. 2013, 4, 2154 10.1038/ncomms3154. 23877168 PMC3759039

[ref21] Chen K.; Dao T. D.; Ishii S.; Aono M.; Nagao T. Infrared Aluminum Metamaterial Perfect Absorbers for Plasmon-Enhanced Infrared Spectroscopy. Adv. Funct. Mater. 2015, 25, 6637–6643. 10.1002/adfm.201501151.

[ref22] Yokoyama T.; Dao T. D.; Chen K.; Ishii S.; Sugavaneshwar R. P.; Kitajima M.; Nagao T. Spectrally Selective Mid-Infrared Thermal Emission from Molybdenum Plasmonic Metamaterial Operated up to 1000 °C. Adv. Funct. Mater. 2016, 4 (12), 1987–1992. 10.1002/adom.201600455.

[ref23] Yoo D.; Vidal-Codina F.; Ciracì C.; Nguyen N.-C.; Smith D. R.; Peraire J.; Oh S.-H. Modeling and observation of mid-infrared nonlocality in effective epsilon-near-zero ultranarrow coaxial apertures. Nat. Commun. 2019, 10, 4476 10.1038/s41467-019-12038-3. 31578373 PMC6775091

[ref24] Rodríguez-Cantó P. J.; Martínez-Marco M.; Rodríguez-Fortuño F. J.; Tomás-Navarro B.; Ortuño R.; Peransí-Llopis S.; Martínez A. Demonstration of near infrared gas sensing using gold nanodisks on functionalized silicon. Opt. Express 2011, 19, 7664–7672. 10.1364/OE.19.007664. 21503075

[ref25] Zheng X.; Kupresak M.; Moshchalkov V. V.; Mittra R.; Vandenbosch G. A. A potential-based formalism for modeling local and hydrodynamic nonlocal responses from plasmonic waveguides. IEEE Trans. Antennas Propag. 2019, 67, 3948–3960. 10.1109/TAP.2019.2907807.

[ref26] Zheng X.; Kupresak M.; Mittra R.; Vandenbosch G. A. A boundary integral equation scheme for simulating the nonlocal hydrodynamic response of metallic antennas at deep-nanometer scales. IEEE Trans. Antennas Propag. 2018, 66, 4759–4771. 10.1109/TAP.2018.2851290.

[ref27] Kongsuwan N.; Demetriadou A.; Horton M.; Chikkaraddy R.; Baumberg J. J.; Hess O. Plasmonic nanocavity modes: From near-field to far-field radiation. ACS Photonics 2020, 7, 463–471. 10.1021/acsphotonics.9b01445.

[ref28] Xomalis A.; Chikkaraddy R.; Oksenberg E.; Shlesinger I.; Huang J.; Garnett E. C.; Koenderink A. F.; Baumberg J. J. Controlling Optically Driven Atomic Migration Using Crystal-Facet Control in Plasmonic Nanocavities. ACS Nano 2020, 14, 10562–10568. 10.1021/acsnano.0c04600. 32687323 PMC7458481

[ref29] Zheng X.; Verellen N.; Volskiy V.; Valev V. K.; Baumberg J. J.; Vandenbosch G. A. E.; Moshchalkov V. V. Interacting plasmonic nanostructures beyond the quasi-static limit: a “circuit” model. Opt. Express 2013, 21, 31105–31118. 10.1364/OE.21.031105. 24514685

[ref30] Zheng X.; Kupresak M.; Verellen N.; Moshchalkov V. V.; Vandenbosch G. A. E. A Review on the Application of Integral Equation-Based Computational Methods to Scattering Problems in Plasmonics. Adv. Theory Simul. 2019, 2, 1900087 10.1002/adts.201900087.

[ref31] Christopoulos T.; Tsilipakos O.; Sinatkas G.; Kriezis E. E. On the calculation of the quality factor in contemporary photonic resonant structures. Opt. Express 2019, 27, 14505–14522. 10.1364/OE.27.014505. 31163898

[ref32] Zheng X.; Verellen N.; Vercruysse D.; Volskiy V.; Van Dorpe P.; Vandenbosch G. A. E.; Moshchalkov V. On the Use of Group Theory in Understanding the Optical Response of a Nanoantenna. IEEE Trans. Antennas Propag. 2015, 63, 1589–1602. 10.1109/TAP.2015.2400471.

[ref33] Hugall J. T.; Baumberg J. J. Demonstrating photoluminescence from Au is electronic inelastic light scattering of a plasmonic metal: the origin of SERS backgrounds. Nano Lett. 2015, 15, 2600–2604. 10.1021/acs.nanolett.5b00146. 25734469 PMC4415038

[ref34] Poggio A. J.; Miller E. K. Chapter 4: Integral equation solutions of three-dimensional scattering problems. Computer Techniques for Electromagnetics 1970, 159–264. 10.1016/B978-0-08-016888-3.50008-8.

[ref35] Chang Y.; Harrington R. A surface formulation for characteristic modes of material bodies. IRE Trans. Antennas Propag. 1977, 25, 789–795. 10.1109/TAP.1977.1141685.

[ref36] Rao S.; Wilton D.; Glisson A. Electromagnetic scattering by surfaces of arbitrary shape. IRE Trans. Antennas Propag. 1982, 30, 409–418. 10.1109/TAP.1982.1142818.

[ref37] Hutchison J. A.; Centeno S. P.; Odaka H.; Fukumura H.; Hofkens J.; Uji-I H. Subdiffraction limited, remote excitation of surface enhanced Raman scattering. Nano Lett. 2009, 9, 995–1001. 10.1021/nl8030696. 19199757

[ref38] Chikkaraddy R.; Singh D.; Pavan Kumar G. Plasmon assisted light propagation and Raman scattering hot-spot in end-to-end coupled silver nanowire pairs. Appl. Phys. Lett. 2012, 100, 043108 10.1063/1.3679649.

[ref39] Huang Y.; Fang Y.; Sun M. Remote excitation of surface-enhanced Raman scattering on single Au nanowire with quasi-spherical termini. J. Phys. Chem. C 2011, 115, 3558–3561. 10.1021/jp109888e.

[ref40] Dasgupta A.; Singh D.; Pavan Kumar G. Dual-path remote-excitation surface enhanced Raman microscopy with plasmonic nanowire dimer. Appl. Phys. Lett. 2013, 103, 151114 10.1063/1.4824896.

[ref41] Zhang Z.; Fang Y.; Wang W.; Chen L.; Sun M. Propagating surface plasmon polaritons: towards applications for remote-excitation surface catalytic reactions. Adv. Sci. 2016, 3, 1500215 10.1002/advs.201500215. PMC5061028 27774380

[ref42] Gargiulo J.; Violi I. L.; Cerrota S.; Chvátal L.; Cortés E.; Perassi E. M.; Diaz F.; Zemánek P.; Stefani F. D. Accuracy and Mechanistic Details of Optical Printing of Single Au and Ag Nanoparticles. ACS Nano 2017, 11, 9678–9688. 10.1021/acsnano.7b04136. 28853862

[ref43] Nedev S.; Urban A. S.; Lutich A. A.; Feldmann J. Optical Force Stamping Lithography. Nano Lett. 2011, 11, 5066–5070. 10.1021/nl203214n. 21992538 PMC3833036

[ref44] Urban A. S.; Lutich A. A.; Stefani F. D.; Feldmann J. Laser Printing Single Gold Nanoparticles. Nano Lett. 2010, 10, 4794–4798. 10.1021/nl1030425. 20957994

[ref45] Chikkaraddy R.; Turek V. A.; Kongsuwan N.; Benz F.; Carnegie C.; van de Goor T.; de Nijs B.; Demetriadou A.; Hess O.; Keyser U. F.; Baumberg J. J. Mapping Nanoscale Hotspots with Single-Molecule Emitters Assembled into Plasmonic Nanocavities Using DNA Origami. Nano Lett. 2018, 18, 405–411. 10.1021/acs.nanolett.7b04283. 29166033 PMC5806994

[ref46] Lin K.-Q.; Yi J.; Zhong J.-H.; Hu S.; Liu B.-J.; Liu J.-Y.; Zong C.; Lei Z.-C.; Wang X.; Aizpurua J. Plasmonic photoluminescence for recovering native chemical information from surface-enhanced Raman scattering. Nat. Commun. 2017, 8, 14891 10.1038/ncomms14891. 28348368 PMC5379060

[ref47] Benz F.; Chikkaraddy R.; Salmon A.; Ohadi H.; De Nijs B.; Mertens J.; Carnegie C.; Bowman R. W.; Baumberg J. J. SERS of individual nanoparticles on a mirror: size does matter, but so does shape. J. Phys. Chem. Lett. 2016, 7, 2264–2269. 10.1021/acs.jpclett.6b00986. 27223478 PMC4916483

[ref48] Woessner A.; Lundeberg M. B.; Gao Y.; Principi A.; Alonso-González P.; Carrega M.; Watanabe K.; Taniguchi T.; Vignale G.; Polini M.; Hone J.; Hillenbrand R.; Koppens F. H. L. Highly confined low-loss plasmons in graphene–boron nitride heterostructures. Nat. Mater. 2015, 14, 421–425. 10.1038/nmat4169. 25532073

[ref49] Pons-Valencia P.; Alfaro-Mozaz F. J.; Wiecha M. M.; Biolek V.; Dolado I.; Vélez S.; Li P.; Alonso-González P.; Casanova F.; Hueso L. E.; Martín-Moreno L.; Hillenbrand R.; Nikitin A. Y. Launching of hyperbolic phonon-polaritons in h-BN slabs by resonant metal plasmonic antennas. Nat. Commun. 2019, 10, 3242 10.1038/s41467-019-11143-7. 31324759 PMC6642108

[ref50] Epstein I.; Alcaraz D.; Huang Z.; Pusapati V.-V.; Hugonin J.-P.; Kumar A.; Deputy X. M.; Khodkov T.; Rappoport T. G.; Hong J.-Y.; et al. Far-field excitation of single graphene plasmon cavities with ultracompressed mode volumes. Science 2020, 368, 1219–1223. 10.1126/science.abb1570. 32527826

