# Supplementary material for: Interfering Plasmons in Coupled Nanoresonators to Boost Light Localization and SERS
Source: Nano Lett. 2021 Mar 11;21(6):2512–8. doi: 10.1021/acs.nanolett.0c04987 (PMC7995252; doi:10.1021/acs.nanolett.0c04987)
Supplement: Supplementary file 1 — nl0c04987_si_001.pdf [file nl0c04987_si_001.pdf]

Supplementary Information for

Interfering Plasmons in Coupled Nano-resonators to Boost Light Localisation and SERS

Angelos Xomalis [1], Xuezhi Zheng [1,2], Angela Demetriadou [3], Alejandro Martínez [4], Rohit Chikkaraddy [1], Jeremy J. Baumberg [1*]

[1] NanoPhotonics Centre, Cavendish Laboratory, Department of Physics, JJ Thompson Avenue, University of Cambridge, Cambridge, CB3 0HE, United Kingdom
[2] Department of Electrical Engineering (ESAT-TELEMIC), KU Leuven, Kasteelpark Arenberg 10, BUS 2444, 3001 Leuven, Belgium
[3] School of Physics and Astronomy, University of Birmingham, Birmingham B15 2TT, United Kingdom
[4] Nanophotonics Technology Center, Universitat Politècnica de València, Valencia 46022, Spain

Supplementary Text

S1 Mid-infrared characterization of the disk array

Reflectance spectra of plasmonic disk arrays are shown in the mid-infrared regime (Fig. S1). We measured two disk diameters 6 µm (green) and 6.5 µm (red) to show the tuning clearly. Theoretical predictions are obtained with a full-wave solver (Lumerical) and experimental measurements with the aid of a quantum cascade laser (Laser Tune, Block Engineering) with operation wavelength between 6.5 - 12.5 µm (1600 – 800 cm^{-1}), tunable pulse durations between 20 to 300 ns and a repetition rate up to 3 MHz. The infrared light is focused onto the sample with a reflective objective (x40, 0.5 NA) resulting in a focal spot of ~10 µm. The reflection from the sample is redirected with a ZnSe beamsplitter to a mercury-cadmium-telluride infrared detector. We studied the visible resonances of the µ-resonators in the main text (Fig. 2c) and in section S4.

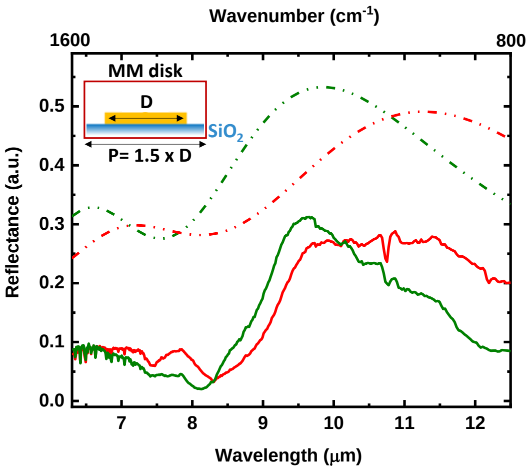

Fig. S1: Reflectance spectra of a plasmonic disk array on SiO_2 substrates. Reflectivity theoretical predictions (dashed) and experimental measurements (solid) for disk resonators. Green (red) colors correspond to 6 µm (6.5 µm) disk diameter (D). The period of the array is P = 1.5 x D in all cases.

S2 Full-wave simulations of different excitation sources

Full-wave simulations (Lumerical) comprising plane-wave and Gaussian beam sources (Fig. S2) are used to model the experimental conditions. In NPoMs (black, Fig. 2a), extreme light confinement exceeds $E/E_0 >$ 200 with two dominant resonances (10) and (20) for a nanoparticle facet size of 20 nm [1, 2]. To better understand the influence of the µ-resonator on light localization, we perform simulations where we consider a bare disk of 6 µm diameter and record the near-field at the center and 5 nm above the surface (red, Fig. S2a). Full analysis of the disk modes is in section S4. We also simulate the near-field of NPoR constructs (dark red). In all cases the nanoparticle is stationary at the disk center with a dielectric spacer of 1.3 nm. The superposition of NPoM and disk modes delivers near-field as high as $E/E_0 \sim 800$ for a wavelength of 750 nm. Thus, it is clear that the high-order modes of the disk drive light localization in the nanocavity. Here we consider no facet for the nanoparticle in the NPoR construct for simplicity.

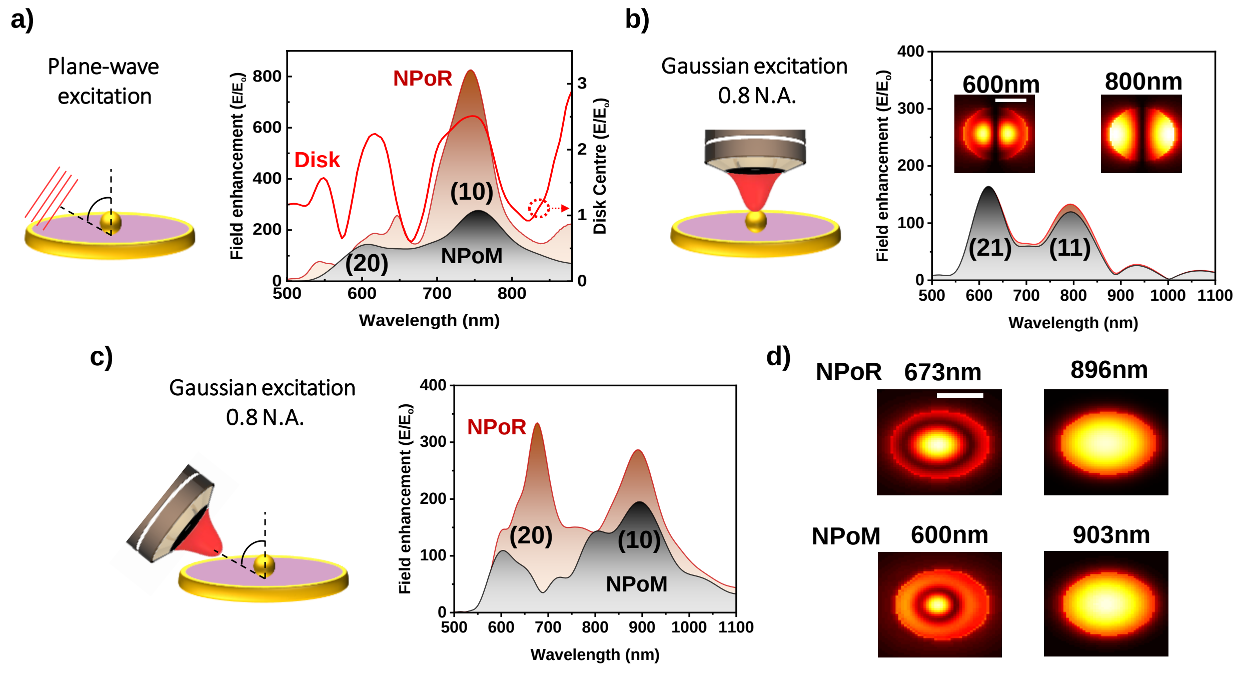

Figure S2. Light confinement of NPoR for plane-wave and Gaussian excitation: a) Simulated near-field of NPoR (dark red), NPoM (black), and bare disk (red) in the gap for plane-wave excitation at 52°. b) Field enhancement for 0.8 NA Gaussian beam at normal incidence. Inset field maps correspond to cross-section along the gap. c) Excitation with Gaussian beam at an angle of 52°. d) Field map cross-sections of c) show modes of NPoR and NPoM plasmonic constructs in the gap. In all cases, the dielectric gap is 1.3 nm thick, disk diameter is 6 µm and scale bar is 10 nm.

As discussed in the main text, experimental findings show slightly reduced SERS contrast compared to theoretical predictions (Fig. S2a). One factor is that plane-excitation, used in our theoretical models, does not correspond to experimental excitation conditions. Consequently, here we examine excitation with high NA Gaussian beams and nanoparticle facets of 20 nm. In all simulations, the excitation point and nanoparticle position is at the center of the µ-resonator. For normal incidence, field enhancement is E/E_0 ~180 for both plasmonic constructs at a pump wavelength of 633 nm (Fig. S2b). Near-field maps shows that a Gaussian source at normal incidence cannot excite (10) and (20) modes but (21) and (11) instead (see inset, Fig. S2b) [3]. The reduced light localization can be explained if we consider an ideal Gaussian beam passing through a linear polarizer and then focusing through a high NA objective. In this case, the phase across the focal region has opposite sign (Gouy phase), meaning that opposite beam

points on the back focal plane of the objective correspond to opposite phase resulting in cancellation of each other when interfering at the focal point (E_z=0, while E_x and E_y survive). One way to overcome this issue experimentally is to use a radial polarizer before the objective lens. Fortunately, real nanoparticle facets are not perfectly symmetric (containing triangle shape facets and small apexes) thus polarization along the z-axis (E_z) survives, giving the SERS signals. This case is not quite our experimental findings, as it gives similar near-fields for NPoR and NPoM constructs (experimental SERS of NPoRs is threefold higher compared to NPoMs). Lastly, we investigate a combination of the two previous cases: oblique excitation (52°) with a Gaussian beam (Fig. S2c). Here light localization is higher in NPoRs compared to NPoMs comprising the same (10) and (20) resonances (Fig. S2d). This example is able to reproduce the modes expected and shows a near-field of NPoRs >3x times higher compared to NPoMs at the pump wavelength (633 nm).

S3 Description of the Computational Method

Our simulations are obtained using an in-house solver based on the Boundary Element Method (BEM) algorithm [4, 5]. The BEM algorithm uses the homogeneous space Green function and the planar multilayer Green function, so that NPoRs (modelled as a faceted nanoparticle on top of a disk) and NPoMs (modelled as a faceted nanoparticle on top of an infinitely large mirror) can be appropriately described. To demonstrate the validity of the solver, we compare total scattering intensities from a NPoR plasmonic system for the BEM solver and a full-wave solver (Lumerical) as the nanoparticle is placed at different radial positions on disk (Fig. S3). Throughout our theoretical discussions, we assume a time harmonic dependence $e^{-i\omega t}$.

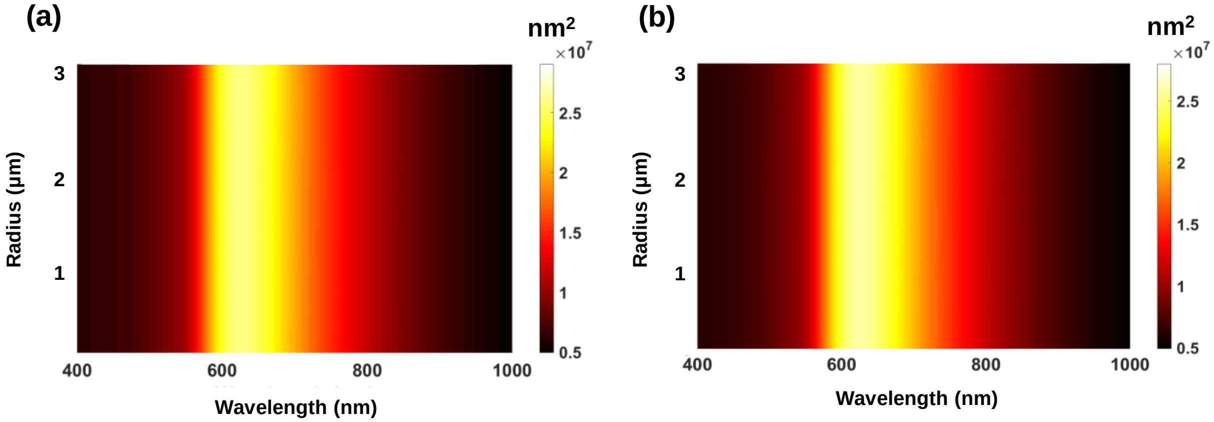

Fig. S3.1: Simulated scattering intensity as the nanoparticle placed at different radial positions onto disk. (a,b) Simulations obtained with (a) the full-wave solver (Lumerical) and (b) as calculated with BEM. In all cases, we consider 80 nm Au nanoparticles with 20 nm facet size and 100 nm thick Au disk with 6 µm diameter excited by plane waves (polarized along the gap) incident at an angle of 52°.

The details of the algorithm and the implementation of our in-house solver can be found in [4, 5]. For any given system (e.g. NPoR or NPoM constructs) and at a specific wavelength (or frequency), the main equation that we solve is

$$\mathbf{Z} \cdot \mathbf{I} = \mathbf{V}. \tag{1}$$

In (1), $\mathbf{Z}$ stands for the system matrix which describes the complete electromagnetic behavior of the system; $\mathbf{V}$ is the excitation vector which describes how an incident field couples to the system; and $\mathbf{I}$ is

the equivalent source vector which is the source of the scattered waves (i.e., the secondary waves). Here we use the eigenvalue problem of (1),

$$\mathbf{Z} \cdot \mathbf{I}_n = \lambda_n \cdot \mathbf{I}_n. \tag{2}$$

where $\mathbf{I}_n$ is the nth eigenmode of the system and λ_n the corresponding eigenvalue. Clearly, both are independent of excitation. To evaluate how well a mode is coupled with an incident field, we introduce the so-called coupling efficiency,

$$\langle \mathbf{I}_n, \mathbf{V} \rangle = \int_B \mathbf{I}_n \cdot \mathbf{V} ds. \tag{3}$$

The coupling efficiency is defined as the projection (i.e., the dot product) of the excitation vector onto the nth eigenmode. The integration is done with respect to the boundary of the system, B.

BEM simulations record the scattering intensity as the diameter of the disk increases from 1 to 6 µm. As expected from their relative areas, the scattering intensity of the NPoR becomes over two orders of magnitude larger than the NPoM (black).

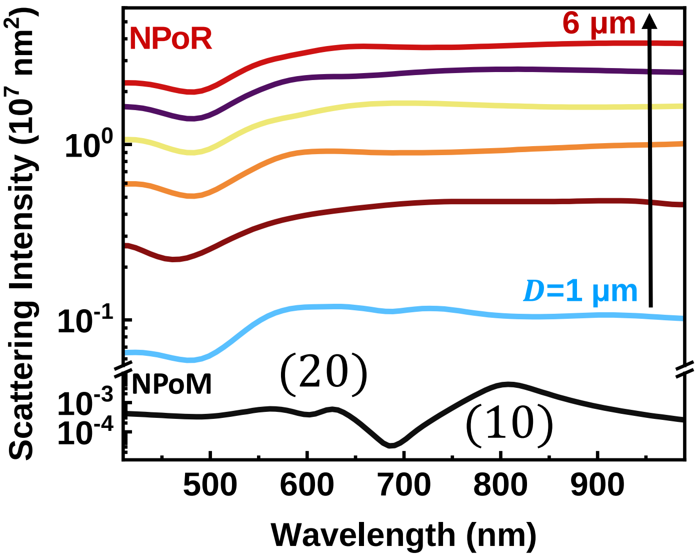

Fig. S3.2: BEM simulated scattering intensity of NPoR and NPoM plasmonic constructs**.** Total scattering intensity NPoR (light blue to red) for increasing disk diameters (D=1-6 µm) and NPoM (black) under plane-wave excitation at optimal incident angle of 52°. The diameter of the Au nanoparticle is 80 nm and facet size 20 nm. The thickness of the Au disk is 100 nm.

Here we solve the eigenvalue problem in (2) for the NPoM system. The following data provide a complementary view of Fig. 2a.

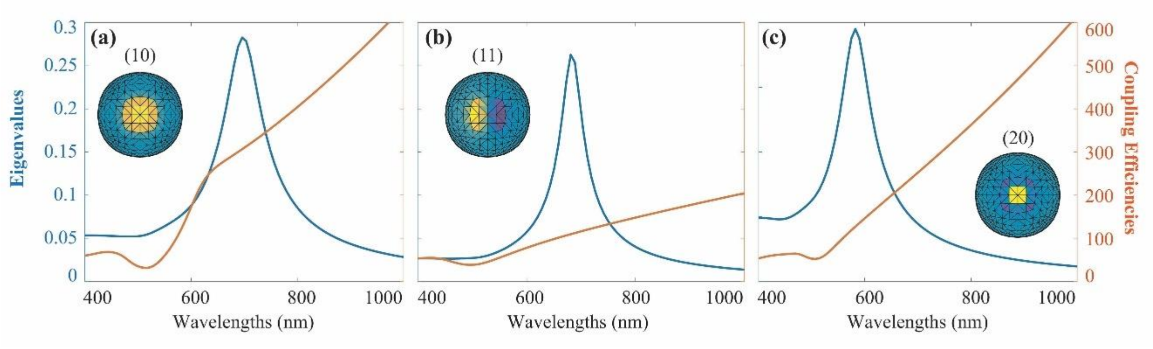

Fig. S3.3: Eigenvalues, eigenmodes and coupling efficiencies of NPoM construct. (a-c) Eigenvalues (blue) and coupling efficiencies (orange) of the (10) (a), (11) (b) and (20) (c) modes. Insets show the surface charge distributions of the modes (bottom view at the nanogap facet). Blue (yellow) codes for negative (positive) charge. The coupling efficiency is calculated for a p polarized plane-wave excitation at optimal incident angle of 52°.

In the following, we solve eigenvalue problem in (2) for the NPoR system. Briefly, our main equation is

$$\begin{pmatrix} \mathbf{Z}_{pp} & \mathbf{Z}_{pd} \\ \mathbf{Z}_{dp} & \mathbf{Z}_{dd} \end{pmatrix} \begin{pmatrix} \mathbf{I}_p \\ \mathbf{I}_d \end{pmatrix} = \begin{pmatrix} \mathbf{V}_p \\ \mathbf{V}_d \end{pmatrix}. \quad (4)$$

In (4), $\mathbf{Z}_{pp}$, $\mathbf{Z}_{pd}$, $\mathbf{Z}_{dp}$ and $\mathbf{Z}_{dd}$ stand for the matrices describing the self-coupling of the particle, the mutual coupling between particle and disk, the coupling between disk and particle, and the self-coupling of the disk, respectively. $\boldsymbol{V}_p$ and $\boldsymbol{V}_d$ are the excitation vectors describing the incident field at the position of the nanoparticle and the disk. $\boldsymbol{I}_p$ and $\boldsymbol{I}_d$ are the equivalent sources induced at the nanoparticle and the disk. Instead of directly solving the eigenvalue corresponding to the entire system in (4), we focus on the nanoparticle and transform (4) into

$$\mathbf{Z}'\mathbf{I}_p = \mathbf{V}'. \quad (5)$$

In (5), we define

$$\mathbf{Z}' = \mathbf{Z}_{pp} - \mathbf{Z}_{pd}\mathbf{Z}_{dd}^{-1}\mathbf{Z}_{dp}, \ \mathbf{V}' = \mathbf{V}_p - \mathbf{Z}_{pd}\mathbf{Z}_{dd}^{-1}\mathbf{V}_d. \quad (6)$$

The eigenvalue problem for (5) reads

$$\mathbf{Z}'\mathbf{I}'_n = \lambda'_n\mathbf{I}'_n. \quad (7)$$

The coupling efficiency is written as

$$\left\langle \mathbf{I}'_n, \mathbf{V}' \right\rangle = \int_B \mathbf{I}'_n \cdot \mathbf{V}' ds. \quad (8)$$

We solve (7) and (8) for the NPoR construct. The following data provide a complementary view to Fig. 2b.

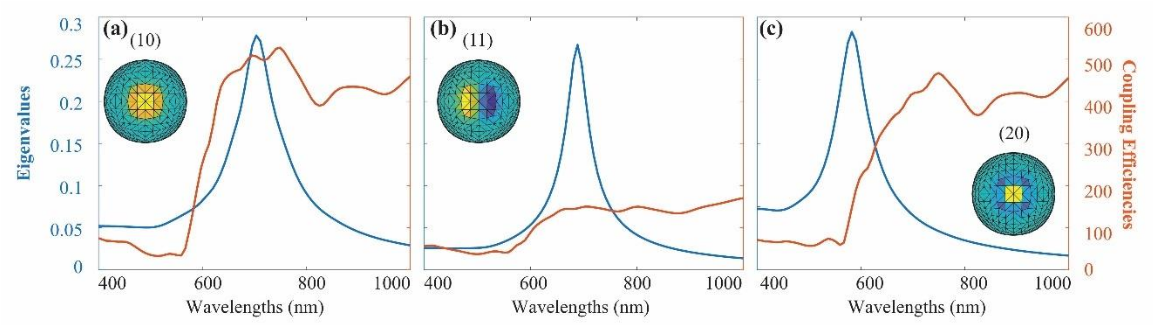

Fig. S3.4: Eigenvalues, eigenmodes and coupling efficiencies of NPoR construct: (a-c) Eigenvalues (blue) and coupling efficiencies (orange) of the (10) (a), (11) (b) and (20) (c) modes. Insets show the surface charge distributions of the modes (the bottom view). Blue (yellow) stands for negative (positive) charge. The coupling efficiency is calculated for a p polarized plane-wave excitation at optimal incident angle of 52°.

S4. Resonances in the Disk

To model the disk modes for a finite thickness d, we use an approach similar to [6] by assuming that the resonances are due to the reflection of SPPs at the boundary of the disk. We consider a disk (with permittivity ε_1) immersed in a homogeneous environment of permittivity ε_2 and that the center of the disk is the origin of the employed coordinate system. Inside the disk, the z component of the electric field (E_z) takes the following form,

$$E_{1z}(\rho, z) = a(z)\left[H_m^{(1)}(k_{spp}\rho) + rH_m^{(2)}(k_{spp}\rho)\right]e^{im\varphi}. \tag{9}$$

In (9), $a(z)$ is the profile of the SPP along the vertical direction whose detailed form can be found in the Appendix C.2 of [6]. At a specific wavelength, the SPP propagates with a lateral wavenumber k_{spp}. For a given m order, $H_m^{(1)}$ and $H_m^{(2)}$ describe the waves propagating towards the edge and the center where r_m is the reflection coefficient,

$$r_m = \frac{+2\pi\varepsilon_2 k_{spp} H_m^{(1)}(k_{spp}R) I_2 - \left[\partial_{k_{spp}\rho} H_m^{(1)}(k_{spp}\rho)\right]_{\rho=R} \int_{-\infty}^{+\infty} \frac{H_m^{(1)}(k_{2\rho}R)}{\left[\partial_{k_{2\rho}\rho} H_m^{(1)}(k_{2\rho}\rho)\right]_{\rho=R}} I_1(-k_z) I_1(+k_z) k_{2\rho} dk_z}{-2\pi\varepsilon_2 k_{spp} H_m^{(2)}(k_{spp}R) I_2 + \left[\partial_{k_{spp}\rho} H_m^{(2)}(k_{spp}\rho)\right]_{\rho=R} \int_{-\infty}^{+\infty} \frac{H_m^{(1)}(k_{2\rho}R)}{\left[\partial_{k_{2\rho}\rho} H_m^{(1)}(k_{2\rho}\rho)\right]_{\rho=R}} I_1(-k_z) I_1(+k_z) k_{2\rho} dk_z}. \tag{10}$$

In (10), $k_{2\rho}, I_1$and I_2 are defined as

$$k_{2\rho} = \sqrt{k_2^2 - k_z^2}, \tag{11}$$

$$I_1(k_z) = \int_{-\infty}^{+\infty} \varepsilon(z) a(z) e^{ik_z z} dz,\ I_2(k_z) = \int_{-\infty}^{+\infty} \varepsilon(z) a^2(z) dz. \tag{12}$$

In (12), $\varepsilon(z)$ is defined as

$$\varepsilon(z) = \begin{cases} \varepsilon_2,\ z > |d/2| \\ \varepsilon_1,\ z < |d/2| \end{cases}. \tag{13}$$

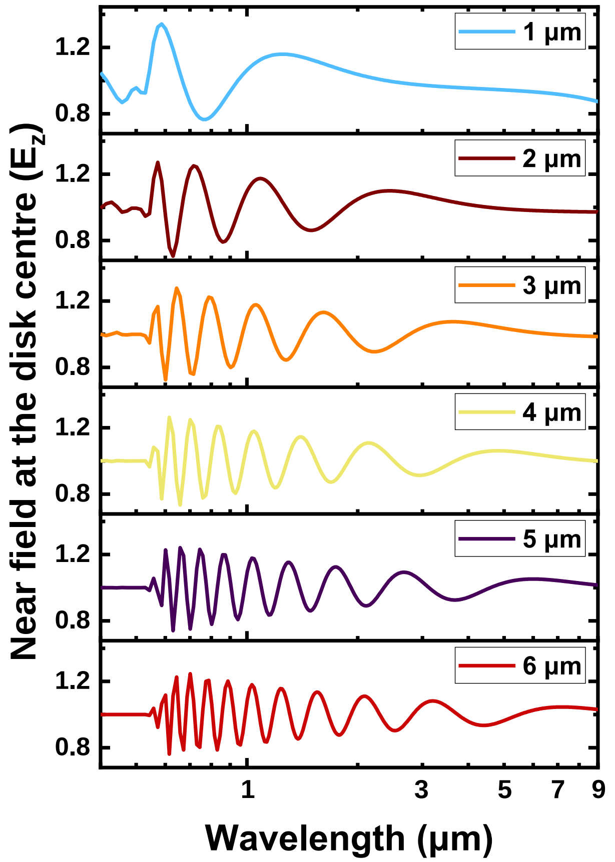

Fig. S4: Near-field (E_z) at centre of the disk for various disk diameter. Calculated near-field at centre of the disk for the odd plasmonic modes of m=1 for a wavelength range 0.4-9 μm.

References

1. N. Kongsuwan, A. Demetriadou, M. Horton, R. Chikkaraddy, J. J. Baumberg, and O. Hess, "Plasmonic nanocavity modes: From near-field to far-field radiation," ACS Photonics **7**, 463–471 (2019).
2. R. Chikkaraddy, X. Zheng, F. Benz, L. J. Brooks, B. De Nijs, C. Carnegie, M.-E. Kleemann, J. Mertens, R. W. Bowman, and G. A. Vandenbosch, "How ultranarrow gap symmetries control plasmonic nanocavity modes: from cubes to spheres in the nanoparticle-on-mirror," ACS Photonics **4**, 469-475 (2017).
3. N. Kongsuwan, A. Demetriadou, M. Horton, R. Chikkaraddy, J. J. Baumberg, and O. Hess, "Plasmonic nanocavity modes: From near-field to far-field radiation," ACS Photonics **7**, 463-471 (2020).
4. X. Zheng, M. Kupresak, R. Mittra, and G. A. Vandenbosch, "A boundary integral equation scheme for simulating the nonlocal hydrodynamic response of metallic antennas at deep-nanometer scales," IEEE Trans. Antennas Propag. **66**, 4759-4771 (2018).

5. X. Zheng, M. Kupresak, V. V. Moshchalkov, R. Mittra, and G. A. Vandenbosch, "A potential-based formalism for modeling local and hydrodynamic nonlocal responses from plasmonic waveguides," IEEE Trans. Antennas Propag. **67**, 3948-3960 (2019).
6. R. Filter, J. Qi, C. Rockstuhl, and F. Lederer, "Circular optical nanoantennas: an analytical theory," Phys. Rev. B **85**, 125429 (2012).
